# Impact of structured sexual health training incorporating standardised patients on health professions students’ sexual health communication competence: A single-blind randomised controlled trial in Tanzania

**DOI:** 10.1371/journal.pone.0360630

**Published:** 2026-10-15

**Authors:** Gift G. Lukumay, Deodatus Kakoko, B. R. Simon Rosser, Michael W. Ross

**Affiliations:** 1 Muhimbili University of Health and Allied Sciences, United Nations Rd, Dar es Salaam, Tanzania; 2 University of Minnesota, Minneapolis, Minnesota, United States of America; University of Turin, ITALY

## Abstract

**Aim:**

To evaluate the effect of a structured sexual health training programme incorporating standardised patients (SPs) on health professions students’ sexual health communication competence, as assessed by SPs, in a Tanzanian setting.

**Design:**

Single-blind randomised controlled trial with parallel-group allocation.

**Setting:**

Muhimbili University of Health and Allied Sciences, Dar es Salaam, Tanzania.

**Participants:**

A total of 312 undergraduate medical and nursing students were enrolled at the university.

**Intervention:**

Structured sexual health training incorporating didactic teaching, small-group discussions, role-play, and SP-based practice sessions.

**Outcome measures:**

Sexual health communication competence was assessed using SP–rated checklists at baseline and at six-month follow-up.

**Results:**

Of 310 health profession students, 73.5% were in their final year. The mean age was 24.29 years (SD = 2.51). 22.6% were female, and 60.0% reported prior sexual health training. Baseline competence scores were similar across groups. At the six-month follow-up, competence improved in both groups, with greater gains in the treatment group. Mixed ANOVA showed a significant time effect (p < .001) and a time-by-group interaction (p = .002). Exploratory item-level analyses indicated higher scores in the treatment group across most sexual health communication competencies, particularly in rapport, empathy, and information-gathering, with no significant difference observed for clear and understandable language.

**Conclusion:**

Structured SP-based sexual health training incorporating SPs significantly improved sexual health communication competence during simulated consultations, particularly in patient-engagement domains. These findings support incorporating culturally adapted sexual health training into healthcare education and using SP assessments to evaluate communication competence in sensitive clinical contexts.

**Trial registration:**

ClinicalTrials.gov, NCT06428058. Registered 30 November 2023.

## Introduction

Sexual health is a vital aspect of individual health and well-being. It is estimated that worldwide, over 1 million curable sexually transmitted infections (STIs) are acquired daily, resulting in more than 374 million new cases each year, including chlamydia, gonorrhoea, syphilis, and trichomoniasis [1]. Beyond STIS, sub-Saharan Africa faces a substantial burden of other sexual and reproductive health issues. These include high rates of unintended pregnancies, teenage childbirth, unsafe abortions, and sexual and gender-based violence [2]. In Tanzania, approximately 35% of sexually active women aged 15–49 reported experiencing symptoms of an STI in 2022. Vulnerable groups, such as adolescent girls, young women, and people with disabilities, face increased risks due to limited access to accurate sexual health information, comprehensive services, and stigma-related barriers within the healthcare system [3].

To overcome ongoing barriers to accessing sexual health services and accurate information, healthcare professionals must possess the skills to communicate effectively and sensitively about sexual health concerns with their clients [4]. Such communication is vital for early detection of sexual health issues and increases the utilisation of preventive and therapeutic measures [4]. However, many healthcare providers report feeling uncomfortable or uncertain when discussing sexual health topics, especially in situations where clients are hesitant to start conversations [5,6]. This discomfort is often tied to limited formal training, personal unease, and cultural sensitivities related to sexuality [6].

In the current Tanzanian undergraduate curricula for medical, midwifery, and nursing programmes, some sexual health content is included but is often presented in a fragmented way [7]. Instructional methods frequently lack practical components necessary to equip students with essential communication and clinical skills [8]. Traditional assessment approaches, such as observing students during clinical rotations, are often insufficient for teaching and evaluating competencies related to sensitive topics, such as sexual health [7,8]. Students may feel inhibited when discussing sexual issues in the presence of faculty, while patients may be reluctant to disclose personal information during observed interactions [8]. Moreover, many preceptors have been trained in curricula that place limited emphasis on sexual health communication skills, which may affect their ability to teach and assess students effectively and consistently [7,9].

To address the gap in healthcare providers’ ability to manage clients’ sexual health concerns effectively, innovative teaching and assessment methods, such as the use of SPs, have been integrated into health professional curricula in many parts of the world [10]. SPs provide a safe, structured, and realistic environment where students can develop and practice essential communication and history-taking skills related to sexual health [11]. This approach allows learners to engage in sensitive conversations without risking harm to real patients [12]. Additionally, SP-based training enables students to make mistakes, receive constructive feedback, and reflect on their communication strategies in a controlled and supportive setting, thereby enhancing their competence and confidence in managing sexual health issues [10,11].

The SP method has been widely adopted in high-income countries to train and assess healthcare professionals in communication and clinical skills [13]. However, its application in low-resource settings, especially within sexual health education, remains limited, despite increasing evidence that SPs improve students’ competence, confidence, and comfort in managing diverse sexual health needs, as well as rating their competence in an appropriate manner [14]. To fill this gap, a structured sexual health curriculum that uses SPs has recently been developed in Tanzania for medical, nursing, and midwifery students [15]. Therefore, this study aimed to evaluate the effect of a structured sexual health training programme incorporating SPs on health professions students’ sexual health communication competence in Tanzania, measured using SP ratings, in a single-blind randomised controlled trial.

## Methods

### Study design

This study employed a single-blind randomised controlled trial (RCT) design to evaluate the effect of a structured sexual health training incorporating SP-based learning on health professions students’ sexual health communication competence in Tanzania. SPs were used in two roles in this study: as part of simulation practice during the intervention and as blind assessors of sexual health communication competence in both the treatment and control groups at baseline and at six-month follow-up. SPs were blinded to health profession students’ group assignments and unaware of whether the health profession students they encountered had received the intervention.

### Study setting

This study was carried out at the Muhimbili University of Health and Allied Sciences (MUHAS) in Dar es Salaam, Tanzania. MUHAS is the oldest and most established public university in the country dedicated exclusively to health sciences. It serves as a key centre for training health professionals across Tanzania and the wider East African region. The university offers a broad array of undergraduate and postgraduate programmes, including medicine, nursing, pharmacy, dentistry, public health, and biomedical sciences.

Recently, the university has led in educational innovation, especially through the development of a sexual health curriculum. This curriculum aims to better prepare future clinicians to address sensitive patient needs [15].

The SP- health profession student encounter took place in a specially designed simulation laboratory at the university. The lab features more than eight private consultation areas, each designed to resemble a real clinical setting. Each space contains two chairs, one for the health profession student and one for the SP, with a table between them. On the table is a tablet that SPs use to assess each health profession student’s interaction immediately after the session, using a structured checklist.

### Inclusion criteria

Participants were eligible if they were medical or nursing students at MUHAS, registered as pre-final year students, and living or studying in Tanzania. Health profession students were also required to have prior clinical experience, defined as at least 100 hours in a clinic, hospital, or community health setting.

### Exclusion criteria

Health profession students were excluded if they were unable to attend all training sessions or the follow-up assessment, or if they were not Tanzanian students.

### Sample size

The sample size was calculated using R for a longitudinal study design with four repeated measurements per participant. The calculation assumed a standardised effect size of 0.57, within-subject variance (σ^2^ = 25.65), between-subject variance (τ^2^ = 2.73), 80% power, and a two-sided significance level of 0.05. Based on these assumptions, a minimum of 281 participants was required to detect a group-by-time interaction, representing differences in the change in communication competence over time between the treatment and control groups. To account for an anticipated attrition rate of 10%, the target sample size was increased to 312 participants, with 156 participants allocated to each study arm

### Study procedures

#### Participant recruitment and randomisation.

Health profession students (medicine and nursing) were recruited from a pool of 820 eligible health profession students via campus flyers and classroom announcements starting on 28th June 2024. To ensure balanced allocation across professional disciplines, medicine and nursing students were randomised separately in batches of ten participants. Randomisation was conducted using a manual envelope-draw method. For each batch, a member of the research team who was not involved in participant recruitment prepared a single opaque, sealed envelope before participant enrolment containing ten folded allocation cards, with five labelled “treatment” and five labelled “control.” Participants independently selected one folded card at random from the envelope without replacement until all ten cards had been drawn, ensuring an equal allocation of five participants to each study arm within each batch.

To maintain allocation concealment, the cards remained unopened until all participants in the batch had completed the selection process, after which group assignments were revealed simultaneously**.** The recruiter had no access to the remaining allocation cards during participant enrolment and did not influence participant allocation. This procedure was repeated until all participants had been assigned to either the treatment or control group.

To maintain the integrity of the outcome assessment, SPs who evaluated the health profession students’ interviews were different from those used during the intervention, and they were completely unaware of the group assignments throughout both the baseline and follow-up assessments.

#### Treatment group.

156 health profession students assigned to the treatment group received structured sexual health training incorporating SP, delivered over four days in September 2024, followed by a one-day boot camp focused on practising sexual health history-taking using SPs. Grounded in the PLISSIT model, the training aimed to enhance sexual health knowledge and communication skills, with particular emphasis on establishing rapport, explaining the purpose of the consultation, ensuring privacy and consent, gathering information effectively, responding empathetically, and closing the session appropriately. Training methods included didactic lectures, small-group discussions, role-plays, and SP simulations addressing common sexual health concerns. In addition to their role in outcome assessment, separate SPs were used within the intervention as part of skills practice during training. To minimise contamination, participants in the treatment group were instructed not to share the intervention materials or discuss the intervention content with those in the control group during the study period.

#### Control group.

156 participants were assigned to the control group, which did not receive the structured sexual health training incorporating SPs. The flow of participants throughout the study, including randomisation and six-month follow-up, is shown in Fig 1.

**Fig 1:**
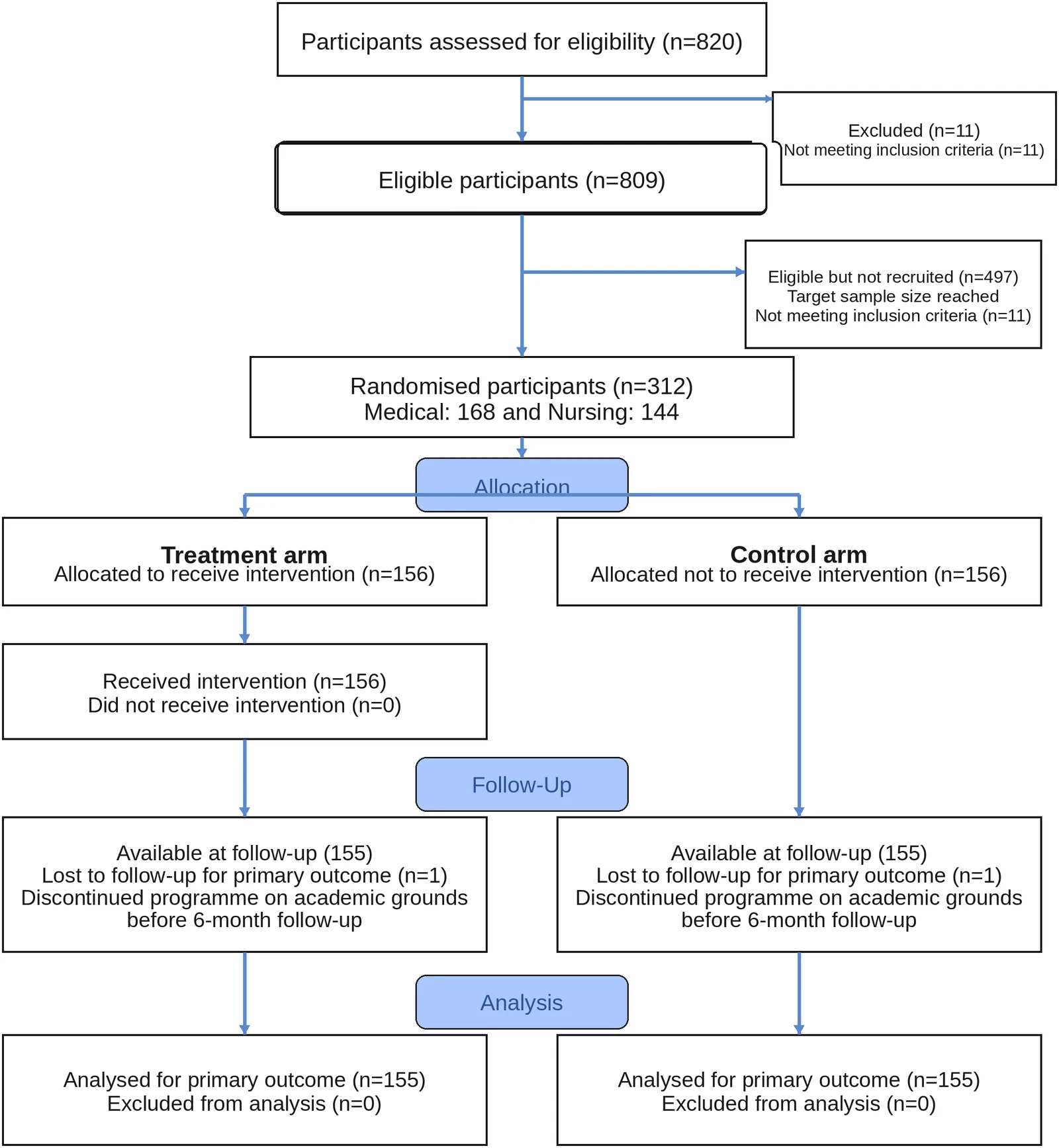
CONSORT Flow Diagram for participants.

### SP recruitment and training

A total of 18 SPs were recruited from the Tanzania School of Performing Arts (TaSUBa) using predefined selection criteria posted on the institution’s internal platform. A recruitment notice detailing each sexual health scenario, including the preferred age and gender for each role, was displayed on the TaSUBa bulletin board. Interested actors accessed a provided link to choose their preferred scenario. Applications were reviewed, and suitable candidates were selected.

Selected SPs participated in a two-day training programme led by MUHAS faculty with expertise in sexual health and prior experience in SP methodology at Johns Hopkins University. The training adhered to the Association of SP Educators (ASPE) Standards of Best Practice and was delivered in Swahili to ensure cultural sensitivity. Day 1 covered programme orientation, psychological safety, scenario familiarisation via table reads, and initial rehearsals. Day 2 focused on checklist and rating training, extended rehearsals, and calibration sessions to ensure consistent, standardised portrayals among SPs assigned to the same scenario. Following the initial training, all SPs engaged in structured pilot simulation sessions to assess readiness for standardised case portrayal and assessment accuracy, in line with ASPE Standards. Each SP participated in pilot encounters with four health professions students, and all interactions were video recorded in Swahili. SPs independently rated health profession students’ sexual communication performance using study-specific assessment tools. Faculty facilitators then conducted individual video reviews and calibration sessions with each SP, comparing SP ratings with faculty observations to identify and resolve discrepancies. This process reinforced agreement between SP ratings and faculty assessments and discussed areas of divergence to ensure consistent interpretation of rating criteria. This iterative feedback was conducted one-on-one with each SP to ensure clarity, reliability, and standardisation. Pilot encounters and calibration sessions were repeated until each SP achieved at least 85% agreement with expert faculty ratings, which was the predetermined competency criterion for participation in the study. All assessment SPs completed the same training and calibration process before data collection. Pilot encounters and calibration sessions were repeated until each SP achieved at least 85% agreement with expert faculty ratings, which was the predetermined competency criterion for participation in the study. Six SPs achieved this criterion after four rehearsal cycles, while the remaining twelve required five cycles before participating in the main study. SPs received daily remuneration for training and simulation sessions, along with transportation allowances to and from MUHAS.

### Simulation scenarios

1. **Domestic and Sexual Violence Case** – A 37-year-old married woman presenting with injuries from intimate partner violence, concerns about repeated STIs, and fear of her husband’s behaviour.
2. **Recurrent STI in a Young Male** – A 25-year-old male student and DJ presenting with a thick penile discharge, dysuria, multiple sexual partners, concern about HIV risk, and relationship strain with his permanent girlfriend.
3. **Adolescent Masturbation Concern** – A 19-year-old male student brought for concerns after being caught masturbating, seeking clarification around sexual development, cultural messages, and emotional well-being.
4. **Painful Intercourse in a Newly Married Woman** – A 25-year-old woman experiencing dyspareunia for one year, relationship tension, fear of medical causes, and difficulty conceiving.
5. **Erectile Dysfunction Case** – 42-year-old adult male presenting with difficulty achieving or maintaining an erection, experiencing psychosocial stressors and relational tension.
6. **Early pregnancy/sugar daddy case** – A 16-year-old adolescent presenting with concerns about vaginal bleeding after having unprotected sex a few months ago. She reported that she is in a relationship with a sugar daddy who is like her uncle. She had a positive pregnancy test three days ago and is now experiencing bleeding. “Aliyah” comes to the clinic scared and unsure of her steps.

### Data collection and timeline

For outcome assessment, all health profession students in both groups completed two interviews with different SPs at baseline, before any training was delivered. The SPs rated health profession students’ communication competence using structured checklists. Each interview lasted up to 10 minutes and was followed by a 5-minute assessment of sexual health communication competence, also rated by the SP using standardised checklists. At baseline, each health profession student (regardless of treatment or control group) completed two interviews with different SPs, yielding 624 student-SP encounters, each of which was rated by the SP.

After completing the structured sexual health training programme incorporating SPs and the six-month follow-up period, all participants conducted a simulated sexual health interview with a SP for the post-intervention assessment. Each health profession student conducted two interviews with different SPs, resulting in two additional SP-student encounters, which the SPs rated again. The SPs remained blind to health profession student group allocation throughout both phases of data collection. The SPs used for assessment were distinct from those involved in intervention.

### Assessment and rating scales

Sexual health communication competence among health profession students was assessed using a 10-item SP assessment checklist covering key interpersonal communication skills. The checklist comprised the following items: C1, Introduction and role clarification; C2, Elicitation of additional concerns; C3, Respectful and professional manner; C4, Appropriate questioning technique; C5, Summarisation and clarification; C6, Active listening; C7, Emotional support and empathy; C8, Exploration of patient worries and expectations; C9, Clear and understandable language; and C10, Consultation closure and next steps.

The assessment checklist was adapted from previously published communication assessment instruments and underwent content review by six clinicians and faculty members with expertise in sexual health, clinical supervision, and health professions education, including four from Muhimbili University of Health and Allied Sciences (MUHAS), one from the University of Minnesota, and one from Johns Hopkins University. Feedback from the expert panel informed refinements to the checklist before pilot testing. The revised checklist was then pilot tested with 24 health profession students not included in the main study. In the present study, the checklist demonstrated good internal consistency (Cronbach’s α = 0.86).

Each checklist item was scored on a three-point scale (0 = not performed, 1 = partially performed, 2 = performed), yielding a total score ranging from 0 to 20 for each SP encounter. At both baseline and the six-month follow-up, each participant completed two SP encounters. The total scores from the two encounters were averaged to generate a single participant-level competence score for each assessment time point. These participant-level mean scores served as the dependent variable in the mixed repeated-measures ANOVA.

To minimise recall effects, different SP scenarios were used at baseline and follow-up. All scenarios were developed from the same blueprint to ensure comparable learning objectives, communication tasks, and levels of difficulty, and were randomly assigned to participants at each assessment. The study outcome and statistical analysis were prespecified in the study protocol. Each encounter was independently assessed by a single calibrated SP using the standardised assessment checklist, and ratings were entered directly into a Qualtrics database via structured observation protocols.

### Ethical considerations

#### Ethics approval and consent to participate.

This study was conducted through a collaboration between Muhimbili University of Health and Allied Sciences (MUHAS) and the University of Minnesota (U of M). Ethical approval was secured from the Tanzania National Institute for Medical Research’s Institutional Review Board (NIMR/HQ.R.8c. Vol.IX/4487, approved on 30 November 2023), which granted full ethical clearance, and from the University of Minnesota Institutional Review Board (Protocol ID: STUDY00019076). The U of M IRB classified the study as qualifying for Common Rule Exemption Category 2, applicable only within the U.S. The study was registered prospectively at ClinicalTrials.gov (NCT06428058).

All participants and SPs received detailed information about the study and signed informed consent before any procedures began. Participation was completely voluntary, and participants were assured they could withdraw at any time without penalty. To safeguard privacy, all data were handled confidentially, and personal identifiers were removed during analysis.

#### Inclusivity in global research.

Additional information regarding the ethical, cultural, and scientific considerations specific to inclusivity in global research is included in the Supporting Information (S1 Checklist).

### Data analysis

The analysis was conducted as a complete-case analysis. Participants with missing 6-month outcome data were excluded. Descriptive statistics were calculated to summarise participant characteristics, including gender, discipline (nursing or medicine), and year of study. Continuous variables were presented as means and standard deviations, and categorical variables as frequencies and percentages. A mixed-design repeated-measures analysis of variance (ANOVA) was conducted to assess changes in overall communication competence over time between the treatment and control groups. Time (baseline versus six-month follow-up) was treated as the within-subjects factor, and group (treatment versus control) as the between-subjects factor. Effect sizes were reported using partial eta squared (η²p). In addition, a linear mixed-effects model was fitted to the encounter-level data, with participant included as a random intercept and SP scenario as a fixed effect to account for repeated observations within participants and potential variation in scenario difficulty. Prior to hypothesis testing, the assumptions of repeated-measures ANOVA were evaluated. Normality of residuals was assessed using the Shapiro–Wilk test and by inspecting residual plots. Homogeneity of variance was assessed using Levene’s test, and influential observations were evaluated using boxplots of standardised residuals. Although the communication competence score was bounded between 0 and 20, repeated-measures ANOVA was considered appropriate because residual plots showed no substantial departures from normality, no extreme outliers were identified, and the primary findings were confirmed by the linear mixed-effects model. Detailed results of the assumption checks are presented in S1 Table.

At the item level of communication competence, Mann–Whitney *U* tests were used to compare the treatment and control groups at the six-month follow-up. Item-level analyses were considered exploratory, and *p*-values were adjusted for multiple comparisons using the Holm procedure. Effect sizes were calculated as the standardised effect size (*r* = *Z*/√*N*) for each Mann–Whitney U test. All statistical analyses were performed using IBM SPSS Statistics version 26.0. All statistical tests were two-tailed, and a *p*-value of <0.05 was considered statistically significant

## Results

We recruited 312 health profession students from a pool of 820 eligible candidates. Of the 312 randomised participants, 310 (99.4%) completed the 6-month follow-up and were included in the complete-case analysis. One participant from each study group was excluded after being disqualified from their academic programme before follow-up. The sample included a slightly higher proportion of medical students (53.5%, n = 166) than nursing students. Most participants (73.5%, n = 228) were finalists, either in their fifth year of medical studies or their fourth year of nursing studies. Approximately one-quarter of participants were female (22.6%, n = 70). Additionally, more than half (60%, n = 186) reported having received some form of sexual health training before this study. The mean age of participants was 24.29 years (SD = 2.51). (See Table 1)

**Table 1:** Baseline Demographic Characteristics of Participants.

Characteristics	Treatment n (%)	Control n (%)	Total n (%)
**Discipline**			
Medical	83(53.5)	83 (53.5)	166(53.5)
Nursing	72(46.5)	72(46.5)	144(46.5)
**Year of Study**			
Final	114(73.5)	114(73.5)	228(73.5)
Penultimate	41(26.5)	41 (26.5)	82(26.5)
**Gender**			
Female	32(20.6)	38 (24.5)	70(22.6)
Male	121(78.1)	117 (75.5)	238(76.8)
Other/Prefer Not to Answer	2(1.3)	0 (0.0)	2(0.6)
**Prior Sexual Health Education**			
Yes	92(59.4)	94 (60.6)	186(60.0)
No	51(32.9)	48 (31.0)	99(31.9)
Other/Prefer Not to Answer	12(7.7)	13 (8.4)	25(8.1)

### Sexual health communication competence among health profession students

At baseline, the mean sexual health communication competence score was similar between the control group (12.89, SD = 3.42) and the treatment group (12.86, SD = 3.21). By follow-up, competence scores increased in both groups, with the control group reaching a mean of 14.74 (SD = 3.51) and the treatment group 16.23 (SD = 2.69) (see Table 2).

**Table 2:** Sexual health communication competence score at baseline and six-month follow-up by participants’ group.

Group	Time point	N	Mean (SD)
Control	Baseline	155	12.89(3.42)
	Follow-up	155	14.74(3.51)
Treatment	Baseline	155	12.86(3.21)
	Follow-up	155	16.23(2.69)

### Effect of the intervention on sexual health communication competence among health profession students

There was a significant main effect of time, F (1, 308) = 113.61, p < .001, partial η^2^ = 0.27, indicating a substantial improvement in sexual health communication competence from baseline to six months across both groups. Importantly, the time-by-group interaction was also statistically significant, F (1, 308) = 9.64, p = .002, partial η^2^ = 0.03, indicating that participants who received the intervention demonstrated greater improvements in sexual health communication competence over time than those in the control group, and the estimated between-group difference in change was 1.52 points (95% CI, 0.56 to 2.48; *p* = 0.002). (see Table 3).

**Table 3:** Effect of the intervention on sexual health communication competence among health profession students.

Effect	df	F	p-value	Partial η²	95% CI
Time	1,308	113.61	<0.001	0.269	–
Time x Group	1,308	9.64	0.002	0.030	1.52 (0.56-2.48)

The effect estimate and 95% confidence interval represent the between-group difference in change from baseline to follow-up and were obtained from a linear regression model of the change score**.**

An additional linear mixed-effects model, fitted to account for repeated observations within participants and differences among SP scenarios, produced consistent findings, with the group × time interaction remaining statistically significant (F (1, 903.09) = 17.90, *p* < 0.001).

### Interaction plot of sexual health communication competence scores over time by groups

Fig 2 illustrates the divergence between the control and treatment groups: the treatment group showed a steadily rising trajectory from baseline to six months, whereas the control group rose more slowly.

**Fig 2:**
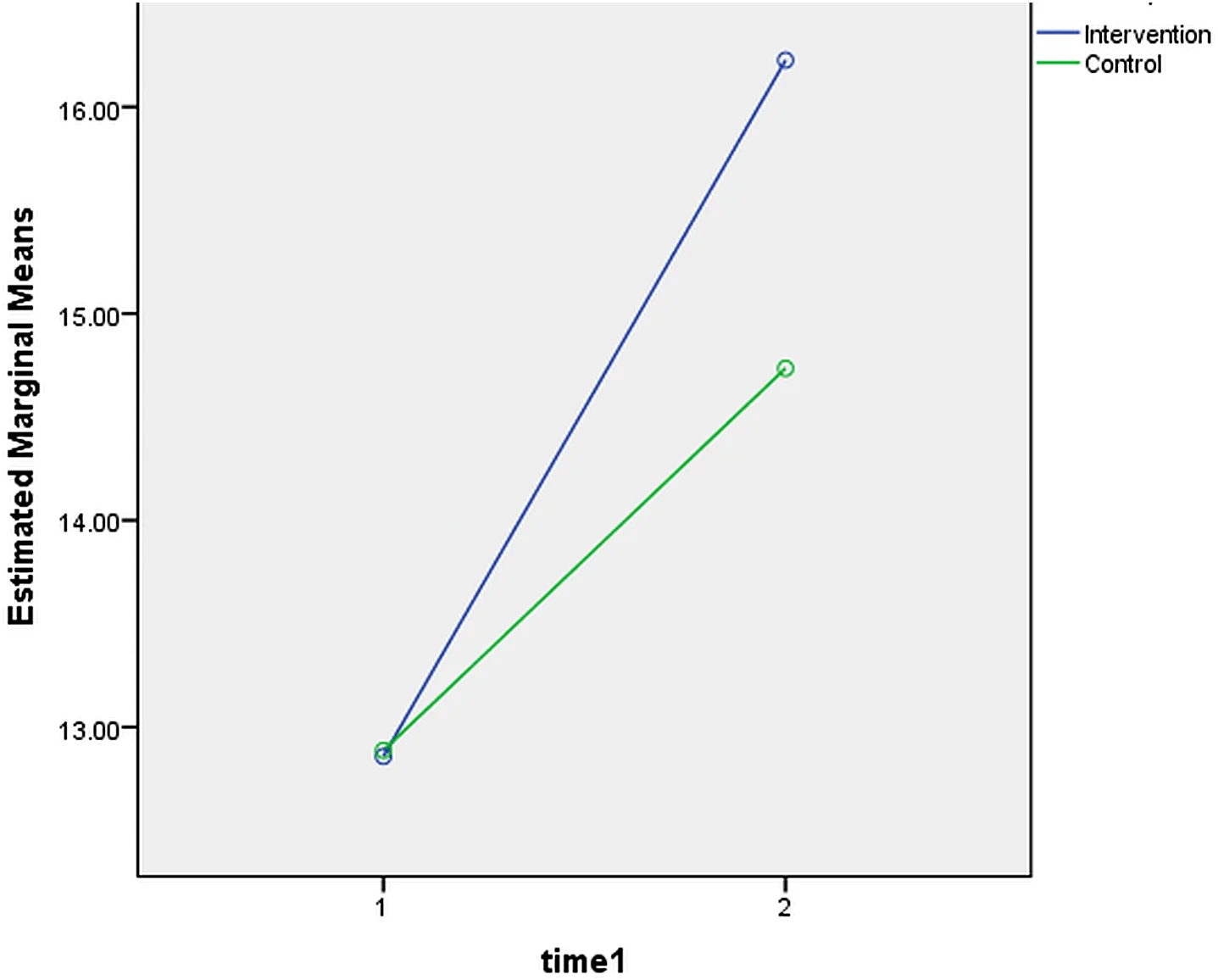
Interaction plot of SHCC scores over time by treatment and control groups.

Interaction plot illustrating mean SHCC scores at baseline and six-month follow-up for treatment and control groups.

### Item-level sexual health communication competence at six-month follow-up

An exploratory item-level analysis showed that participants in the treatment group demonstrated significantly higher competence scores than controls on most communication competence items at six-month follow-up. Statistically significant differences were observed for items C1–C8 and C10 (all Holm-adjusted *p* < 0.05), with treatment participants consistently achieving higher mean scores. No statistically significant difference was observed for item C9 (*p* = 0.160). Effect sizes for the significant item-level comparisons were small (see Table 4)

**Table 4:** Item-level sexual health communication competence at six-month follow-up.

Item	Communication Competence Domain	Control Mean (SD)	Treatment Mean (SD)	*Holm-adjusted p-value†*	*Effect size (r)*
C1	Introduction and role clarification	1.48 (0.43)	1.67 (0.38)	<.001	0.23
C2	Elicitation of additional concerns	1.28 (0.56)	1.48 (0.47)	.002	0.18
C3	Respectful and professional manner	1.69 (0.36)	1.83 (0.25)	.001	0.20
C4	Appropriate questioning technique	1.37 (0.46)	1.50 (0.43)	.018	0.14
C5	Summarization and clarification	1.15 (0.61)	1.42 (0.54)	<.001	0.22
C6	Active listening	1.69 (0.32)	1.79 (0.29)	.006	0.16
C7	Emotional support and empathy	1.47 (0.45)	1.65 (0.37)	.001	0.20
C8	Exploration of PT worries and expectations	1.19 (0.64)	1.51 (0.49)	<.001	0.26
C9	Clear and understandable language	1.89 (0.21)	1.93 (0.19)	.160	0.8
C10	Consultation closure and next steps	1.42 (0.53)	1.57 (0.44)	.016	0.14

† Exploratory between-group comparisons were performed using the Mann–Whitney U test. P-values were adjusted for multiple comparisons using the Holm procedure. Effect sizes are reported as standardized effect sizes (*r* = *Z*/√*N).*

## Discussion

This study evaluated the effect of a structured sexual health curriculum that incorporated SP simulations on health professions students’ sexual health communication competence in Tanzania. Students who received the intervention showed significantly greater improvements in sexual health communication competence during simulated consultations than those in the control group. These findings suggest that the intervention improved SP-rated sexual health communication competence in simulated consultations.

To the best of our knowledge, this is the first randomised controlled study in Tanzania and possibly throughout sub-Saharan Africa to rigorously evaluate the effectiveness of a structured sexual health training programme incorporating SPs in developing sexual health communication competence among health professions students, with competence assessed by SPs. While SP-based sexual health training is well established in high-income countries and has been linked to increases in confidence, communication, and handling sensitive conversations [16–18], such approaches have rarely been tested in low-resource settings. The structured sexual health training incorporating SPs was culturally adapted to the Tanzanian context while incorporating internationally recognised frameworks, such as the PLISSIT model [15]. This blend of local relevance and evidence-based structure made the intervention both contextually appropriate and academically sound.

An exploratory item-level analysis suggested that improvements were most noticeable in competencies related to rapport, respect, and empathy, as well as information gathering and patient engagement. This suggests that the intervention primarily improved interpersonal and exploratory communication behaviours demonstrated during simulated sexual health consultations, which are important components of effective sexual health communication. These findings align with recent evidence showing that simulation-based and SP–based training significantly boost health profession students’ communication skills and clinical interactions [19,20].

While the treatment group experienced notably greater improvements compared to the control group, scores for sexual health communication competence also increased in the control group over the six months. This rise might be due to practice effects from repeated SP encounters, ongoing clinical rotations where both groups participate, and natural maturation as clinical experience grows. Although participants in the treatment group were advised not to share materials or discuss the intervention with the control group during the study, we cannot entirely exclude informal information exchange. The greater improvements seen in the treatment group indicate that the intervention offered advantages beyond standard educational activities.

In contrast, no significant improvement was observed in competencies related to communication clarity, particularly in the use of clear, understandable language (C9). This pattern may reflect the influence of sociocultural norms. In the Tanzanian context, explicit Swahili terminology for sexual health topics may be seen as sensitive or socially inappropriate, causing health profession students to use indirect or euphemistic language. While such strategies might align with cultural expectations [6] they could compromise clarity and limit measurable gains in this competency when assessed using structured checklists.

A natural question arises: can we genuinely rely on SPs, especially in LMICs, to assess health profession students’ sexual health communication competence? This is a valid concern, mainly because evidence on the use of SPs in sexual health, particularly in LMICs, is limited. However, it is important to note that our SPs received extensive training, not only in performing their roles but also in evaluating health profession students’ competencies using structured checklists. Additionally, inter-rater reliability between SPs and faculty has been studied in LMICs [11], with results showing acceptable agreement. In fact, in sensitive areas like sexual health, SP assessments may better reflect real interactions, as health profession students tend to be more open when instructors do not directly observe them.

In general, SP provides a unique opportunity to practice patient-centred communication competencies in a controlled setting, enhancing both verbal and nonverbal communication skills [21]. In this way, students rarely had the opportunity to practice these conversations in a safe, judgment-free environment. These findings align with previous studies conducted in high-income settings [13,22,23], but they are especially meaningful here because they demonstrate that these techniques can be just as effective when adapted to African contexts.

A key characteristic of this intervention is its cultural adaptation to the Tanzanian context, reflected in the curriculum development process, the simulated case scenarios, the language used during training, and the backgrounds of the SPs. The use of SP in the sexual health curriculum in this study was not merely imported or adopted; instead, it was constructed with local relevance in mind. This makes the success of this SP-based sexual health training not just a scientific finding, but a significant step forward in decolonising sexual health education and making it more inclusive and effective for all learners.

## Conclusion

This study demonstrated that structured SP-based sexual health training incorporating SPs significantly improved health profession students’ sexual health communication competencies during simulated consultations. Students who received the intervention showed greater improvement over time than those in the control group, suggesting an added benefit of targeted SP-based sexual health communication training.

These findings support the incorporation of structured SP-based sexual health training into healthcare curricula to strengthen students’ sexual health communication competencies. They also support the use of SP assessments as a structured method for evaluating these competencies in simulated, culturally sensitive clinical scenarios.

### Strengths

A key strength of this study is its longitudinal randomised controlled design, with health profession students assessed at baseline and at six-month follow-up. This design enables evaluation of changes in sexual health communication competence over time and provides evidence of sustained improvement after the intervention. The observed differences between the intervention and control groups at follow-up support the effectiveness of the structured sexual health training programme incorporating SPs beyond routine learning

Another strength is the use of SPs as blinded assessors of health profession students’ sexual health communication performance. This approach enabled assessment of both the content and delivery of communication, capturing not only what health profession students asked but also how they engaged with patients. SP ratings enhanced consistency across encounters while maintaining a high level of realism in the simulated clinical interactions, thereby strengthening the validity of the assessment of sexual health communication competence.

### Limitations

Several limitations should be considered. Firstly, item-level analyses of sexual health communication competence were exploratory and involved multiple comparisons; therefore, these findings should be interpreted with caution. Secondly, sexual health communication competence was evaluated using SP encounters, which, although structured and consistent, may not fully represent communication behaviours in real clinical settings and may be subject to some degree of rater variability.

The study was conducted at a single institution, which may limit the generalisability of the findings to other settings. Additionally, although the six-month follow-up enabled evaluation of sustained improvement, the long-term effects of the intervention on clinical practice and patient outcomes were not assessed. Finally, sociocultural influences on sexual health communication, particularly language use in sexual health discussions, may have affected how certain competencies were expressed and evaluated.

## Supporting information

S1 Table Results of assumption checks for the repeated-measures ANOVA. (DOCX)

S2 Checklist Inclusivity in Global Research Checklist. (DOCX)

## Abbreviations

ICS: interpersonal communication skills
LMICs: low- and middle-income countries
MHTA: medical history-taking ability
MUHAS: muhimbili university of health and allied sciences
RCT: randomised controlled trial
SP: standardised patients
STIs: sexually transmitted infections.
