# Supplementary material for: Impact of structured sexual health training incorporating standardised patients on health professions students’ sexual health communication competence: A single-blind randomised controlled trial in Tanzania
Source: PLoS One. 2026 Oct 15;21(10):e0360630. doi: 10.1371/journal.pone.0360630 (PMC13671498; doi:10.1371/journal.pone.0360630)
Supplement: Supplementary file 1 [file pone.0360630.s001.docx]

**Supplementary Table S1. Assessment of the assumptions underlying the repeated-measures ANOVA**

| **Assumption** | **Assessment method** | **Result** |
| --- | --- | --- |
| Normality | Shapiro–Wilk test of standardised residuals | Baseline: *p* = 0.001; Follow-up: *p* < 0.001 |
| Homogeneity of variance | Levene's test | Baseline: *p* = 0.543; Follow-up: *p* = 0.070 |
| Influential observations | Boxplots of standardised residuals | Three mild outliers at baseline; two mild outliers at follow-up; no extreme outliers |
